# A microemulsion co-loaded with Schizandrin A–docetaxel enhances esophageal carcinoma treatment through overcoming multidrug resistance

**DOI:** 10.1080/10717544.2016.1225854

**Published:** 2017-02-03

**Authors:** Xiangyu Su, Chanchan Gao, Fangfang Shi, Xiaoyao Feng, Lin Liu, Ding Qu, Cailian Wang

**Affiliations:** 1 Department of Oncology, Zhongda Hospital, School of Medicine, Southeast University, Nanjing, P.R. China,; 2 Affiliated Hospital of Integrated Traditional Chinese and Western Medicine, Nanjing University of Chinese Medicine, Nanjing, P.R. China, and; 3 Jiangsu Province Academy of Traditional Chinese Medicine, Nanjing, P.R. China

**Keywords:** Docetaxel, drug resistance, esophageal carcinoma, microemulsion, Schizandrin A

## Abstract

Multidrug resistance (MDR) is the major underlying cause of the low 5-year survival rate of esophageal carcinoma. In this study, we developed a novel microemulsion system (SD-ME) co-loaded with docetaxel (DTX) and Schizandrin A, a potent chemotherapeutic agent and a potential drug resistance modulator, respectively. In the physicochemical characterization studies, SD-ME displayed a well-defined spherical shape and size (56.62 ± 4.16 nm), a narrow polydispersity index (PDI, 0.132 ± 0.002), and a negative surface charge (−19.81 ± 3.11 mv). In the cellular uptake studies, SD-ME with a DTX concentration of 30 μg/mL exhibited a 3.9-fold enhancement of DTX internalization in DTX-resistant EC109 (EC109/DDR) cells in comparison to that observed for EC109 cells, and the mechanisms were associated with reducing P-gp expression and inhibiting P-gp ATPease. The half-maximal inhibitory concentrations (IC_50_) of DTX and SD-ME against EC109/DDR cells were 40.57 ± 0.39 and 3.59 ± 0.06 μg/mL, respectively. Likewise, the apoptotic rate of EC109/DDR treated with SD-ME increased up to 20-fold compared to that observed with free DTX. In anticancer efficacy studies *in vivo*, SD-ME markedly retarded the tumor growth of nude mice bearing EC109/DDR tumor xenografts compared with D-ME and free DTX throughout the duration of study. Consequently, mice treated with SD-ME had the highest survival rate (37.5%) during the observation period (70 days). In addition, there were no apparent side effects after the administration of SD-ME. Overall, our study provides evidence for SD-ME as an effective drug delivery system for enhanced MDR tumor treatment.

## Introduction

Esophageal carcinoma (EC), a malignant neoplasm with high incidence, is the sixth leading cause of cancer-related mortality in the world (Tew et al., 2005). About 456 000 new cases are reported every year worldwide, and nearly 80% of cases occur in the developing countries. Its incidence is 3-fold higher in men than in women (Ferlay et al., 2015). Surgery is the most effective method of treatment for the early-stage disease (stage I). However, surgery is also effective in patients with early resectable esophageal cancers, without evidence of metastatic disease, and those patients might be cured if complete resection is achieved (Nomura et al., 2016).

Chemotherapy is the major treatment option for stage IV EC patients who are diagnosed with metastasis in other organs or who cannot tolerate surgery. Docetaxel (DTX) is a commonly used chemotherapeutic drug for the treatment of esophageal cancer (Miyawaki et al., 2016) in addition to its clinical applications in the treatment of cancers of the lung (Pereira et al., 2013), head (Kogashiwa et al., 2010), ovary (Sorbe et al., 2013), breast (Nakatsukasa et al., 2016a,b), and prostate (Xiao et al., 2016). However, owing to the lack of tumor targeting and the fact that DTX is a P-glycoprotein (P-gp) substrate, systemic adverse effects and multidrug resistance (MDR) are commonly observed after a few courses of treatment (Gottesman et al., 2002), leading to premature termination of chemotherapy and consequently failure of anticancer treatment. In the last few decades, many reports have suggested that nano-medicinal strategies are capable of overcoming or averting MDR (Markman et al., 2013; Wang et al., 2015,2016), enhancing therapeutic efficacy (Xu et al., 2016), and reducing side effects (Puvvada et al., 2015; Strong & West, 2015) through an inherent inhibition of the P-glycoprotein (P-gp) and enhanced permeability and retention effect (EPR). These specific properties of nano-medicinal approaches result in greater accumulation of the drug delivery system in the vicinity of tumor sites instead of nonselective distribution. However, until now, only few commercial nanomedicines have been successfully developed for both effective anti-MDR and tumor-specific accumulation. The lack of success in developing commercial nanomedicines for EC likely was attributed to two main reasons. The stability or release of drug delivery *in vivo* is the first reason. The advantages of tumor-specific accumulation and reduction of P-gp-mediated efflux would be attenuated if the structure of the drug delivery system was unstable or if too much drug was released prior to the arrival of the drug delivery system at the targeted sites. Second is the lack of a rational combination in a multicomponent drug delivery system. Conventional drug delivery, without co-loading of a P-gp inhibitor along with the active pharmaceutical, shows only a limited enhancement of the cellular uptake of the drug. Such enhancement is derived from nanomedicine averting the drug efflux mainly through excipient-mediated tight junction opening or by escaping recognition by P-gp, rather than via P-gp inhibition. We propose that combining a potent P-gp inhibitor with a chemotherapeutic agent will lead to synergic anticancer treatment.

Microemulsion (ME), a nano-sized drug delivery system with structural stability, comprises an oil phase, a surfactant, and a co-surfactant. By right of outstanding integration of multicomponent, ME is an appropriate vehicle for the co-delivery of various types of functional agents, resulting in the integration of the respective advantages. For instance, Qu and coworkers developed an ME-based co-delivery system carrying coix seed oil, etoposide, and coix seed polysaccharide, which enhanced apoptosis of tumor cells and improved tumor-specific targeting (Qu et al., 2015,2016). Similarly, incorporation of an anticancer drug and a P-gp inhibitor into a microemulsion system can potentially overcome the MDR in esophageal carcinoma. Nevertheless, studies from Chavanpatil and coworkers (2006) suggested that anticancer agents internalized into the cytoplasm are still susceptible to P-gp-mediated efflux even when co-loaded with verapamil (a classical P-gp inhibitor) because of the variability in the release performance and the effective dose between the therapeutic agents and the efflux inhibitor. Evidently, screening for an appropriate combination of efflux inhibitor and anticancer drug with similar effective concentration and release behavior is of great importance in reversing MDR. Schizandrin A, a hydrophobic extract from a traditional Chinese medicine tonic, has shown a strong P-gp inhibition at a concentration of 25 μM (Huang et al., 2008). Coincidentally, in our preliminary experiments, the half-maximal inhibitory concentration (IC_50_) of DTX against resistant EC109 cells was also approximately 25 μM. In addition, both Schizandrin A and DTX are hydrophobic, which makes their assembly in one single drug delivery system feasible.

Herein, we designed a dual-drug microemulsion co-delivery system using a combination strategy of Schizandrin A and DTX (SD-ME), an anticancer drug and a P-gp inhibitor, respectively. This study focused on whether the incorporation of Schizandrin A and DTX improved the anticancer efficacy against drug-resistant EC *in vitro* and *in vivo*. In addition, the efficacy of SD-ME over its single drug-loaded counterpart (D-ME) and the physical mixture of Schizandrin A and DTX were evaluated from the perspective of cellular uptake, apoptosis induction, P-gp expression, and *in vivo* safety.

## Materials and methods

### Materials

Schizandrin A (>99%) and DTX (>99%) were purchased from Sigma-Aldrich Co., Ltd. (Poole, UK). Labrafil M CS1944 and Cremophor RH40 were offered by BASF Co., Ltd. (Ludwigshafen, Germany). PEG400 was purchased from Aladdin Chemical Co., Ltd. (Shanghai, China). MTT [3-(4,5-dimethylthiazol-2-yl)-2,5-diphenyltetrazolium bromide] assay was sourced from Amresco (Solon, OH). Water in this study was obtained using a Milli Q-water purification system (Merck-Millipore, Billerica, MA). All other chemicals and solvents were of analytical grade. RPMI 1640 medium, fetal bovine serum (FBS), penicillin–streptomycin solution, and phosphate buffer salt (PBS) were obtained from Hyclone (Thermo Scientific, UT).

### Preparation and characterization of microemulsion

Schizandrin A–docetaxel-loaded microemulsion (SD-ME) was synthesized by single emulsion method as described by previous publication (Qu et al., 2015). First, 5 mg of DTX and 5 mg of schizandrin A were simultaneously dissolved in 200 mg of Labrafil M CS1944 with a strong stirring at 600 rpm using an MYP11-2 homoiothermal magnetic stirrer (MeiYingPu instruments Co., Ltd., Shanghai, China). Next, 180 mg of Cremophor RH40 and 60 mg of PEG400 were successively added to the above-mentioned mixture with further vigorous magnetic stirring at room temperature until no visible undissolved substance. At the end of the mixing, a clear solution was obtained after 2.0 mL of deionized water was slowly dropped into the resulting mixture. Likewise, blank microemulsion or DTX-loaded microemulsion (D-ME) was prepared through the similar method expect feeding corresponding drug.

The drug entrapment efficiency (DEE) and drug loading efficiency (DLE) were analyzed by the following equations: DEE (%) = (C_1 _×_ _V_1_/W_DTX1_) × 100%; DLE (%) = (C_2 _×_ _V_2_/W_DTX2_) × 100%, where C_1_, V_1_, C_2_, and V_2_ represent the drug concentration and the volume of the freshly prepared microemulsion, and the drug concentration and the volume of the freeze-dried microemulsion, respectively. W_DTX1_ and W_DTX2_ are the weight of the feeding drug and the weight of the freeze-dried microemulsion, respectively (Qu et al., 2013). The chromatographic condition of DTX used HPLC (1260 Infinity system; Agilent Technologies, Santa Clara, CA) with a reverse Diamond C18 column (4.6 mm × 150 mm, 5 μm particles, 1.0 mL/min). DTX was quantified by ultraviolet detection at 230 nm, and the mobile phase was a mixture of methanol and water (55/45, v/v). The chromatographic condition of Schizandrin A employed the same HPLC system but assayed at 282 nm, and the mobile phase was a mixture of methanol and water (80/20, v/v) with 0.03% triethylamine. In the process of the measurement, the column temperature was maintained at 30 °C and the sample volume injected was 10 μL.

The average particle size and zeta potential of SD-ME were assayed by dynamic light scattering (DLS) using a Beckman Coulter Particle Analyzer (Fullerton, CA). The freshly prepared samples were diluted 10 times in various aqueous environments (PBS of pH 7.4, artificial gastric juice, artificial small intestinal juice, and plasma) and incubated for 2 h at room temperature. The morphologic image was observed using transmission electron microscopy (TEM, JEOL-100CXII, Tokyo, Japan). At the end of the incubation, 10 μL of SD-ME was deposited onto a carbon-coated copper grid to create a thin film. Next, 2% phosphotungstic acid was counterstained to the film for 5 sec. Finally, the grid was allowed to dry under infrared lamp and observed immediately by TEM.

### *In vitro* release of microemulsion

The *in vitro* release profile of D-ME and SD-ME was evaluated by a classic dialysis method but with some modifications. 2 mL of D-ME and SD-ME was added into a dialysis bag (molecular weight cutoff: 10 kDa), followed by immersion in 200 mL of artificial intestinal juice and gastric juice at 37 °C with stirring at 100 rpm using a dissolution apparatus (ZRS-8G, Shanghai, China), respectively. 0.5 mL of sample was withdrawn and filtered through a polycarbonate membrane filter (0.22 μm of pore size) at each predetermined time interval, followed by replacement with equivalent volume of blank corresponding juice. The concentration of DTX and Schizandrin A was assayed by HPLC using the above-mentioned method.

### Cellular uptake of D-ME and SD-ME

#### Cells culture and the mechanism of drug resistance

The human esophageal squamous carcinoma EC109 cells were purchased from the Cell Bank of the Chinese Academy of Sciences (Shanghai, China). The DTX-resistant EC109 (EC109/DDR) cells were induced by our group. Both two cell lines were cultured in RPMI-1640 medium supplemented with 10% FBS, 100 U/mL penicillin, and 100 μg/mL streptomycin at 37 °C in an atmosphere of 5% CO_2_. Logarithmic growing cells were prepared and were used for the cellular experiments.

The EC109/DDR cells were induced according to the reported method (Wang et al., 2013). The 70–80% confluence adherent EC109 cells were treated with 0.625 μg/mL of DTX for 2 h, followed by washing with PBS thrice and culturing in complete RPMI-1640 medium. After 24 h incubation, the surviving cells were cultured continuously in normal medium for 3 passages. The above-mentioned process was repeated three times. At this time, the cells were exposed to 1.0 μg/mL of DTX for 4 h, and the following process was similar as described above. The EC109/DDR cell line was established until the P-gp expression was significantly higher than EC109 cells using P-gp antibody binding assay kit with Anti-Human CD243 (ABCB1) PE as a P-gp monoclonal antibody (San Diego, CA) (Mo et al., 2011). The P-gp ATPase activity interfered by various formulations was investigated by Pgp-Glo assay system with P-gp (Promega, WI) (Jin et al., 2014).

#### Quantification of intracellular DTX

Both EC109 cells and DTX-resistant EC109 cells were seeded into 24-well plates (Costar, USA) at a density of 1 × 10^5^ cells per well and incubated until 70% cells were adhered. The tested formulations were set as follows: (1) free DTX (suspension), (2) D-ME, and (3) SD-ME, each of the group contained 10, 20, and 30 μg/mL DTX. The cells were incubated with various formulations for 4 h in a cell culture incubator. At the end of the treatment, the cells were then washed with 500 μL of PBS five times, followed by treatment with 200 μL of 0.1% sodium dodecyl sulfate (SDS) cell lysis buffer for 3 min. DTX content in cells was quantified by HPLC and the cell protein was detected using a BCA protein assay kit. The formula of uptake was calculated as follows: uptake = Q_intracellular DTX_/Q_cell protein_, where Q_intracellular DTX_ and Q_cell protein_ represent the amounts of intracellular DTX and cell protein in two cell lines, respectively.

#### Intracellular accumulation of rhodamine 123-labeled microemulsion

As a fluorescent P-gp substrate, rhodamine 123 (R123) is readily effluxed in MDR cells and frequently employed to analyze the P-gp function (Wan et al., 2006). In this study, R123-labeled microemulsion (R123-ME) and R123-labeled Schizandrin A-loaded microemulsion (R123/S-ME) were also prepared to explore whether microemulsion formulation or co-loading with Schizandrin A could enhance the accumulation of R123 in EC109/DDR cells. Free R123 was used as a control group of R123-ME. Prior to the experiment, the cells were cultured in 6-well plates (Costar, USA) at 5 × 10^5^ cells per well for 24 h. And then, the cells were treated with 5 μM of free R123, R123-ME, and R123/S-ME for 3 h. At the end of the treatment, the cells were washed in 500 μL of PBS thrice and observed using fluorescence inverted microscope immediately under the environment of phenol red-free RPMI-1640 medium. Afterward, the cells were trypsinized through ethylenediaminetetraacetic acid (EDTA)-free pancreatin, followed by termination with PBS containing 10% FBS. At this moment, the cell suspensions were collected through fully washing with PBS, harvested in 200 μL of incomplete phenol red-free RPMI-1640 medium, and finally quantified the fluorescence by flow cytometry (Guava 6HT).

#### Cell viability assay

The effects of D-ME and SD-ME on cell viability of EC109 and EC109/DDR cells were determined using the MTT assay. Briefly, EC109 cells and EC109/DDR cells were placed at a density of 8000 cells per well in 96-well culture plates (Costar, USA) and allowed to adhere. The two cell lines were incubated with various concentrations of DTX, blank ME, D-ME, and SD-ME, respectively. After treatment for 48 h, the incubation medium was removed, followed by tinting with PBS containing 5 mg/mL MTT for 4 h in a CO_2_ incubator. The resulting formazan crystals were dissolved in 150 μL of DMSO, and the absorbance at 490 nm was recorded using an ELISA reader (iMark, BioRad680, USA). Cell viability (%) was calculated according to the following formula: Cell Viability (%) = (OD of test group/OD of control group) × 100%. The half-maximal inhibitory concentration (IC_50_) was calculated using SPSS 17.0 software (SPSS Inc., IL).

#### Apoptosis induction

EC109/DDR cells were placed at a density of 5 × 10^4^ cells per well in 24-well culture plates (Costar, USA) for 24 h. After the cells were adhered, D-ME and SD-ME with three concentrations (0.1, 1.0, and 10.0 μg/mL of DTX) were incubated with the cells for 12 h. At the end of the treatment, the cells were rinsed with PBS thrice and collected in a manner of suspension in 200 μL of binding buffer, followed by staining with 5 μL of Annexin V-FITC (20 μg/mL) and 5 μL of propidium iodide (PI, 50 μg/mL) for 30 min in the dark, and then diluting in further 300 μL of binding buffer. The treated cells were immediately assayed using flow cytometry (Guava 6HT).

#### Western blotting analysis

EC109 and EC109/DDR cells were treated with various concentrations of D-ME and SD-ME for 48 h, and then the levels of P-gp and β-actin were measured by western blotting. Total protein from the cells was extracted in RIPA lysis buffer (Beyotime, Nanjing, China) supplemented with protease inhibitor cocktail tablets (Sigma, USA) and quantified using a BCA protein assay kit (Thermo Fisher Scientific Inc., Waltham, MA). A total of 30 μg of protein was separated using 10% sodium dodecyl sulfate–polyacrylamide gel electrophoresis (SDS-PAGE) and transferred to a nitrocellulose (NC) membrane (Millipore, MA). The membrane was blocked with 5% nonfat milk and incubated at 4 °C overnight with the desired primary antibody. After washing three times with phosphate buffer solution and Tween 20 (PBST), the membrane was incubated for 60 min with horseradish peroxidase-conjugated secondary antibody diluted in PBST. Protein bands were visualized using enhanced chemiluminescence (Millipore, USA) and detected using a Bio Imaging System (Clinx, China). The relative protein levels were normalized to β-actin as a loading control.

#### In vivo antitumor efficacy

Animals: BALB/c nude mice (weighing 20 ± 2 g) were purchased from the Silaike Laboratory Animal Co., Ltd. (Shanghai, China). The mice were housed in polypropylene cages under standard laboratory conditions at a temperature of 25 ± 1 °C and a relative humidity of 55 ± 5%. All animals used in this experiment were pathogen-free and allowed free access to food and water. The animal experiments were carried out in accordance with the protocol approved by the animal ethics committee at our institution.

The ability of various formulations to inhibit tumor growth and improve immunity was evaluated using EC109/DDR tumor-bearing mice as an animal model. Mice bearing EC109/DDR xenografts were generated by subcutaneous injection of EC109/DDR cells (2 × 10^6^ per mouse) into the right axilla. The mice were randomly divided into four groups (*n* = 12) on day 10 post-xenograft implantation, and intragastrically administrated with DTX suspension, D-ME, and SD-ME at a DTX dose of 10 mg/kg, respectively. Saline was used as a negative control. Tumor growth was monitored by measuring the perpendicular diameter of the tumor with a caliper. The volumes of tumors were calculated according to the following formula: tumor volume (mm^3^) = 1/2 × length × width^2^. The liver/spleen index was calculated using the following formula: liver/spleen index (mg/g) = the weight of liver or spleen/the weight of body. Plasma tumor necrosis factor-alpha and interleukin-6 levels were detected by enzyme-linked immunosorbent assay (ELISA) kit at 37 °C.

### Statistical analysis

The data are shown as the mean ± standard deviation. Statistical significance is tested by the two-tailed Student’s *t*-test. A *p* value less than 0.05 is considered as statistically significant (marked as *); a *p* value less than 0.01 is considered as extremely significant (marked as **).

## Results and disussion

### Preparation and characterization of SD-ME

Microemulsion has emerged as an attractive drug carrier system (frequently with a size range of 20–100 nm) for the reversal of multidrug resistance, as well as for the incorporation of two or more functional hydrophobic components. In general, a blank microemulsion prepared by conventional single emulsion method usually displays a size of approximately 50 nm. In order to assess the particle size, zeta potential and several other pharmaceutical characteristics, including DEE and DLE, after loading the functional agents, the physicochemical properties of D-ME and SD-ME samples were studied extensively. As shown in Table 1, the particle sizes of D-ME and SD-ME were 52.39 ± 2.78 nm and 56.62 ± 4.16 nm, respectively. Polydispersity index (PDI) values for both were less than 0.15. The zeta potentials of the three types of microemulsion were all moderately negative (−15.0 to −20.0 mv) before and after incorporating the hydrophobic drugs. These results suggested that there was no change in the surface properties after loading Schizandrin A and/or DTX. In addition, a stable DEE and a high DLE are of great importance for manufacturing ME for large-scale practical applications. Therefore, we also evaluated the DEE of Schizandrin A and/or DTX for SD-ME and D-ME, as well as the DLE. As presented in Table 1, the DEE of both Schizandrin A and/or DTX was higher than 90%, indicating that ME is a potent solubilization tool for hydrophobic functional agents, which is consistent with previous reports. Besides, we observed that the total DLE of SD-ME was approximately 2%, which indicates a strong loading capability of the microemulsion (according to the feeding of different components). More importantly, this formulation technique ensured that the DTX concentration in aqueous environment ranged from 0.1 μg/mL to 2.0 mg/mL, making it amenable to further dose regulation in the cellular and animal studies.

**Table 1.** Physicochemical characterization of various types of microemulsion (*n* = 3).

Sample	Size (nm)	PDI	Zeta (mv)	DEE^a^ (%)	DEE^b^ (%)	DLE (%)
ME	48.28 ± 1.93	0.122 ± 0.002	−15.25 ± 2.44	NA	NA	NA
D-ME	52.39 ± 2.78	0.121 ± 0.002	−17.32 ± 3.78	93.43 ± 2.12	NA	0.92 ± 0.07
SD-ME	56.62 ± 4.16	0.132 ± 0.002	−19.81 ± 3.11	95.57 ± 1.53	94.12 ± 2.01	1.92 ± 0.24

ME represents blank microemulsion, DEE^a^ represents DEE of DTX in D-ME and SD-ME, DEE^b^ represents DEE of Schizandrin A in SD-ME.

**Figure 1.**
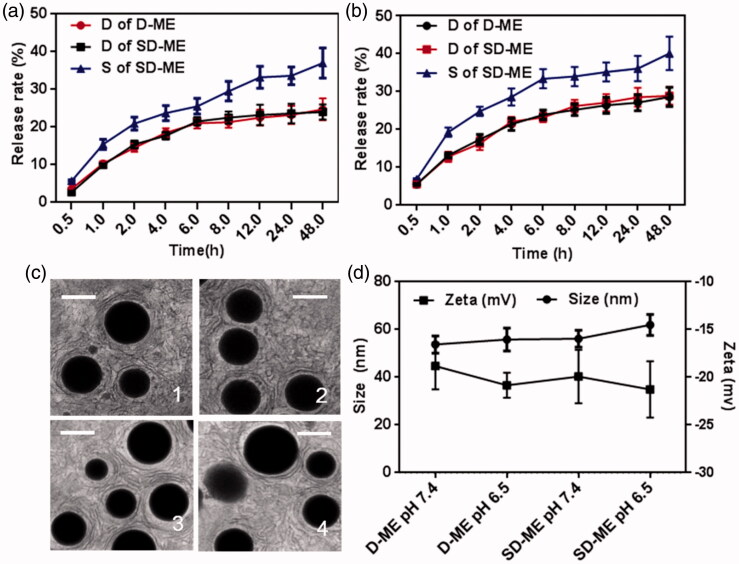
Release profiles, size distribution, and morphology of SD-ME. *In vitro* release profiles of DTX from D-ME and SD-ME and Schizandrin A under (a) artificial intestinal juice and (b) gastric juice; data are presented as mean ± SD (*n* = 3). (c) Morphology of SD-ME in (1) PBS, (2) artificial gastric fluid, (3) artificial intestinal fluid, and (4) 50% FBS observed by TEM. Scale bar represents 50 nm. (d) Changes of size and zeta potential of SD-ME and D-ME under different pH environments for 7 days, data are presented as mean ± SD (*n* = 3); insert pictures are the appearance of SD-ME after storage for 12 h to 7 days.

Drug release plays an important role in the fate of microemulsion *in vivo* as only the drug released from the delivery system exerts the pharmacologic effect. Primarily, we focused on the release rate of the drug and thus evaluated the potential efficiency of drug delivery system. In fact, the release sequence also merits attention as it determines the drug’s intracellular distribution. In order to investigate the potential drug release *in vivo*, we assessed the release profile of Schizandrin A and/or DTX in artificial intestinal fluid and gastric fluid. As shown in Figure 1(a), the 48-h accumulative release of DTX and Schizandrin A from SD-ME in artificial intestinal fluid was 24.6 ± 2.9% and 36.9 ± 4.0%, respectively, which was almost the same as that in PBS pH 7.4 (data not shown). As shown in Figure 1(b), the release behavior in the artificial gastric fluid was similar to that in the artificial intestinal fluid. The 48-h accumulative release of DTX from D-ME and SD-ME was almost the same (approximately 28%), but that of Schizandrin A in SD-ME was obviously higher (40.1%). Because the gastric emptying and the intestinal absorption time are generally not longer than 4 h, we were especially interested in release characteristics at 4 h. Both DTX and Schizandrin A were released less than 20%, in the two artificial fluids, indicating that the majority of the therapeutic agents would be stably encapsulated into the microemulsion after gastric and intestinal digestion or absorption. Notably, Schizandrin A displayed a faster release rate than DTX in both acidic (gastric) or weak base (intestinal) simulations. Such release pattern suggests that Schizandrin A can potentially inhibit the P-gp prior to DTX release in the cytoplasm, and thus effectively enhance the treatment of MDR esophageal carcinoma.

In order to further characterize the structure stability of the drug delivery system in various environments, the morphology of SD-ME was studied using TEM after incubation with 50 times-volume of PBS, artificial intestinal fluid, artificial gastric fluid, and PBS containing 50% (v/v) FBS for 30 min. As shown in Figure 1(c), SD-ME exhibited a nearly perfect spherical appearance with a size of ∼50 nm and a narrow PDI. The fact that the structure was stable under stimulations of pH and protein, even upon 50-fold dilution, indicates that microemulsion was potentially capable of accumulating at the tumor sites through the EPR effect. Furthermore, the 7-day storage stability of SD-ME and D-ME was evaluated by monitoring the changes of particle size and zeta potential. There was no significant variability among different groups. The obtained results showed that SD-ME with a small size and a spherical shape had a strong encapsulating ability and a stable structure that endured various physiological environment stimulations.

### Cellular uptake

**Figure 2.**
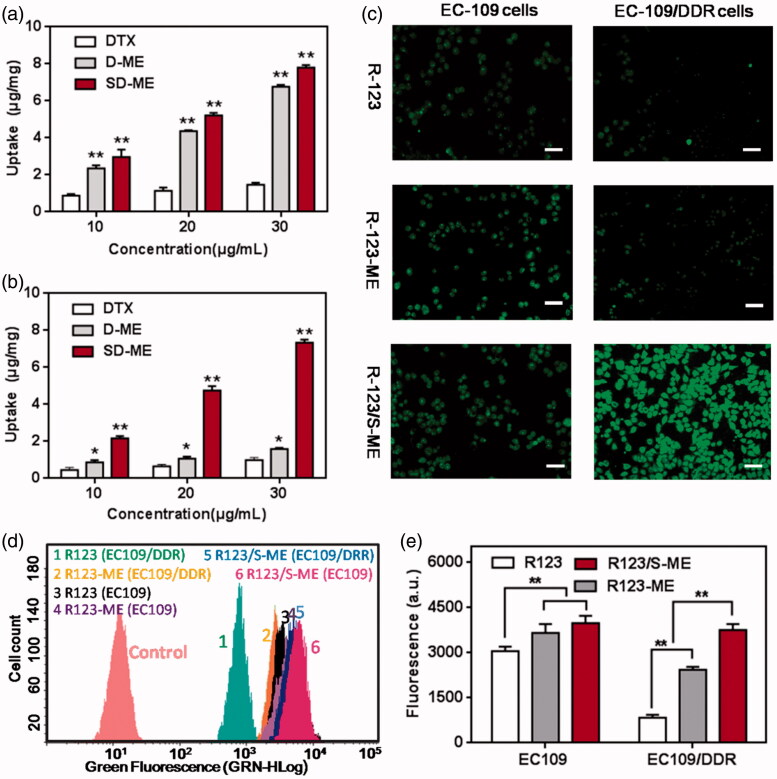
Cellular uptake studies. Intracellular accumulation of DTX in (a) EC109 cells and (b) EC109/DDR cells for 4 h (*n* = 3). **p < 0.01 versus free DTX. (c) Fluorescence images of cells treated with R123, R123-ME, and R123/S-ME. (d) Images of R123 accumulation in EC109 cells and EC109/DDR cells by flow cytometry and (e) corresponding fluorescence quantification (*n* = 3). **p < 0.01.

The enhancement of cellular uptake of the drug formulations in EC109/DDR cells was the most important evaluation criterion. In this study, we compared the difference in cellular uptake between EC109 cells and EC109/DDR cells qualitatively and quantitatively. The cellular uptake of D-ME and SD-ME was evaluated by HPLC. As seen in Figure 2(a), both D-ME and SD-ME achieved a significantly enhanced cellular uptake in EC109 cells compared with free DTX, suggesting that microemulsion formulation indeed promoted the internalization of the drug. However, no significant difference in the cellular uptake of the drug was observed between D-ME and SD-ME, indicating that the incorporation of P-gp inhibitor had limited influence on the endocytosis in non-MDR tumor cells. As expected, the cellular uptake of DTX in EC109/DDR cells was greater for SD-ME than for other preparations presumably because of the microemulsion formulation and the incorporation of Schizandrin A. As depicted in Figure 2(b), the amount of intracellular DTX for SD-ME was 2.01 ± 0.04 μg/mg at a DTX concentration of 10 μg/mL, which was 1.72-fold and 6.93-fold higher than that for D-ME and free DTX, respectively. In further quantitative experiments, R123 (a P-gp substrate) was used as a fluorescence marker because its green fluorescence facilitates the monitoring of cellular uptake. As presented in Figure 2(c), the intracellular fluorescence intensity of EC109 cells after treatment with R123/S-ME was slightly stronger than that for R123-ME and free R123 at a concentration of 5 μM, suggesting that the P-gp inhibitor did not enhance the uptake of EC109 cells to a significant extent. However, when EC109/DDR cells were treated with R123/S-ME for 4 h, the internalized fluorescence was extremely stronger than that for other formulations, further proving the rationale for the incorporation of the P-gp inhibitor and the anticancer agent into one microemulsion. In addition, the R123-labeled cells were trypsinized into a suspension and qualitatively assayed through cytometry. As shown in Figure 2(d and e), the EC109/DDR cellular uptake of R123/S-ME (3751.1 ± 189.6) was 4.5-fold and 1.5-fold higher relative to free R123 and R123-ME, respectively, and consistent with the fluorescence imaging results. Based on the obtained results, the combination of Schizandrin A and DTX into one single system might have the ability to inhibit P-gp-mediated drug efflux.

### Cytotoxicity and evaluation on drug resistance reversal

**Figure 3.**
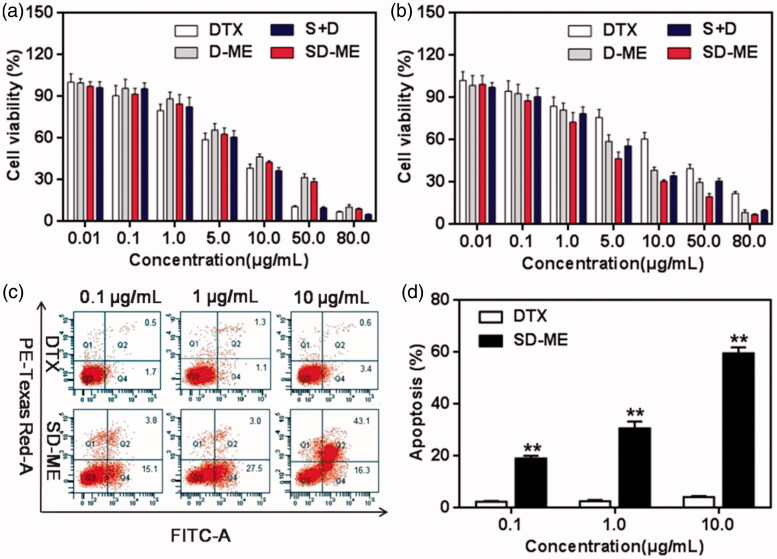
The cytotoxicity of various formulations against (a) EC109 cells and (b) EC109/DDR cells for 48 h (*n* = 6). Study on cell apoptosis induction. (c) The quadrant pictures of EC109/DDR cells treated with DTX and SD-ME at a concentration ranged from 0.1 to 10 μg/mL, and (d) the corresponding quantitative presentation. **p < 0.01 versus free DTX group.

In order to investigate the potential antitumor activity, the anti-proliferative effect of various formulations in EC109 cells and EC109/DDR cells was determined using the classical MTT method. In this experiment, we employed DTX, D-ME, SD-ME, and the physical mixture of Schizandrin A and DTX (S + D) as test groups to evaluate the functional effectiveness of the microemulsion formulation and the drug combination. As shown in Figure 3(a), all the formulations inhibited the growth of EC109 cells in a concentration-dependent manner at the concentration range from 0.01 to 80 μg/mL. The IC_50_ of DTX, D-ME, and SD-ME were 7.52 ± 0.03, 8.93 ± 0.05, and 8.24 ± 0.04 μg/mL, respectively (Table 2). Although DTX displayed slightly greater cytotoxicity than D-ME and SD-ME, there was no statistical difference among all the test groups. However, a greater variability in IC_50_ values was observed when EC109/DDR cells were treated with various formulations for 48 h. As shown in Figure 3(b), DTX only inhibited the growth of less than 40% EC109/DDR cells even at the concentration of 10 μg/mL, suggesting that this MDR tumor cell line was insensitive to chemotherapeutic agents. The formulation of DTX in the microemulsion platform (D-ME group) greatly improved its cytotoxicity in tumor cells. Notably, the IC_50_ of SD-ME decreased up to 3.59 ± 0.06 μg/mL, which was remarkably lower in comparison to that of D-ME. Besides, S + D also displayed a potent ability to reverse drug resistance although its IC_50_ was slightly higher than that of SD-ME, indicating that co-delivery of the anticancer drug and the P-gp inhibitor might be helpful in overcoming MDR. Resistance reversion index (RRI) values shown in Table 2 are indicative of the potential of SD-ME as a promising drug delivery system for addressing the MDR concerns in esophageal carcinoma.

**Table 2.** IC_50_ and resistance reversion index (RRI) of different formulations against EC109 and EC109/DDR cells for 48 h (*n* = 6).

	EC109 cells	EC109/DDR cells	
Formulation	IC_50_ (μg/mL)	IC_50_ (μg/mL)	RRI
DTX	7.52 ± 0.03	40.57 ± 0.39	1.00
D-ME	8.93 ± 0.05	6.96 ± 0.08	5.84
SD-ME	8.24 ± 0.04	3.59 ± 0.06	11.30
S + D	7.48 ± 0.02	5.86 ± 0.10	6.93

^a^RRI = IC_50_ (DTX)/IC_50_ (test samples).

### Cell apoptosis

To investigate the effect of SD-ME on cell apoptosis, three concentration gradients were used to quantify the proportion of apoptotic cells using Annexin V-FITC and PI double staining method. As shown in the quadrant pictures in Figure 3(c), DTX at a concentration ranging from 0.1 to 10 μg/mL induced apoptosis in less than 5% of EC109/DDR cells after a 12-h incubation, probably due to the low cellular uptake resulting from severe drug efflux. However, SD-ME with 0.1 μg/mL of DTX resulted in apoptosis of approximately 19% of EC109/DDR cells. When the concentration of DTX was increased up to 10 μg/mL, the apoptosis induced by SD-ME was increased by 15-fold compared to that of DTX at the equivalent concentration and 3-fold relative to that at a DTX concentration of 0.1 μg/mL. In addition, D-ME exhibited improvement in apoptotic rate compared with DTX in EC109/DDR cells (data not shown), proving the effect of microemulsion formulation on P-gp inhibition. The apoptosis data suggested that SD-ME could promote the apoptosis of EC109/DDR cells through enhanced cellular uptake, as well as rational combination of components.

### Mechanism of drug resistance reversal

**Figure 4.**
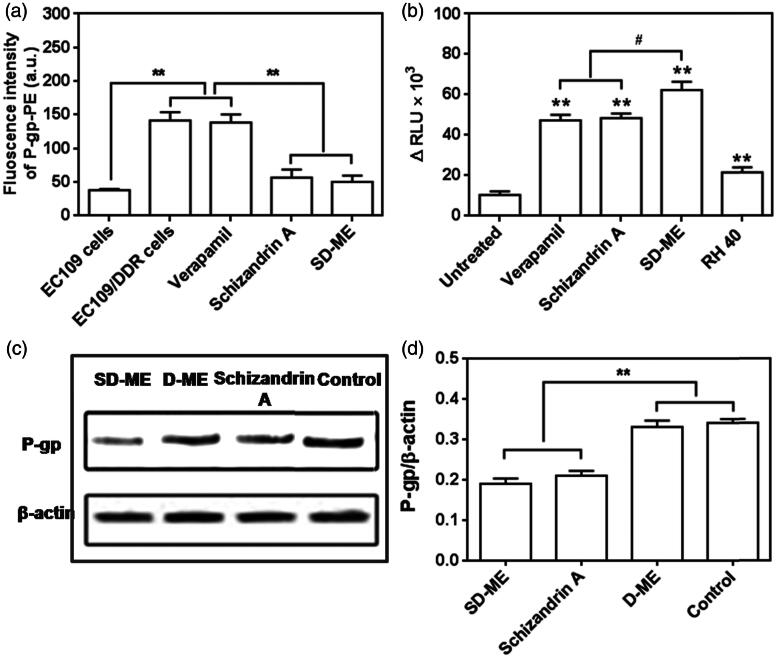
(a) P-gp expression in EC109 cells and EC109/DDR cells with or without incubation of various formulations. (*n* = 4, **p < 0.01 versus EC109 cells) (b) Changes of P-gp ATPase activity stimulated by potential P-gp inhibitor, microemulsion, and surfactant. (*n* = 4, **p < 0.01 versus untreated group; ^#^ *p* < 0.05 versus SD-ME.) (c) Western bolt analysis for P-gp expression treated with Schizandrin A, D-ME, and SD-ME, and (d) the corresponding quantitative analysis. (*n* = 3, **p < 0.01 versus control).

To examine the relationship between the incorporation of Schizandrin A in the microemulsion and the anti-P-gp activity of the resultant formulation, we tested the two potential P-gp inhibition mechanisms: P-gp expression and P-gp ATPase activity, using a P-gp antibody binding assay kit/western blot and the Pgp-Glo assay system, respectively. As shown in Figure 4(a), EC109/DDR cells had a higher P-gp expression than EC109 cells (**p < 0.01), suggesting that drug resistance in EC109 cells was successfully induced. As we expected, the P-gp expression level showed little change after the treatment with 100 μM of verapamil, which was consistent with previous publications (Mo et al., 2011). However, 100 μM of Schizandrin A and SD-ME containing equivalent Schizandrin A remarkably reduced the expression of P-gp in EC109/DDR cells (**p < 0.01 versus verapamil). This result indicated that the anti-resistance effect shown by SD-ME was likely related to the inhibition of P-gp expression by Schizandrin A. Next, we evaluated the effect of SD-ME on the activity of P-gp ATPase in comparison with verapamil, which is a known stimulator of P-gp ATPase activity. In this study, the decrease in the luminescence of untreated samples compared to samples treated with Na_3_VO_4_ represented basal P-gp ATPase activity. Likewise, the difference in the luminescence of formulations-treated samples (relative light unit, ΔRLU) represented the influence of various formulations on P-gp ATPase. The positive ΔRLU value of verapamil (47 321 ± 2719) suggested a strong stimulatory effect on the P-gp ATPase activity. As shown in Figure 4(b), the ΔRLU of Schizandrin A and RH40 were 48 343 ± 2319 and 21 530 ± 2480, respectively, and distinctly higher than the basal P-gp ATPase activity. Assay results indicated that both Schizandrin A and RH40 had a potent capability of reducing the consumption of ATP by P-gp, leading to increased luminescence derived from unconsumed ATP. Consequently, the ΔRLU of SD-ME was determined to be higher than the two P-gp inhibitors (^#^ *p* < 0.05), indicating that the mechanism of P-gp inhibition by SD-ME is likely to involve a synergistic inhibition of the P-gp ATPase activity by Schizandrin A and RH40. Besides, a western blot analysis was also performed to further establish the effects of Schizandrin A and SD-ME on expression levels of P-gp on the surface of EC109/DDR cells. After 48-h incubation of EC109/DDR cells with Schizandrin A and SD-ME, the levels of P-gp expression significantly decreased, but such phenomenon was not observed in the D-ME group (Figure 4c and d). These results indicated that the anti-drug resistance mechanism of SD-ME comprises of dual activities: the reduction of P-gp expression and the inhibition of the P-gp ATPase activity.

### Antitumor efficacy *in vivo*

**Figure 5.**
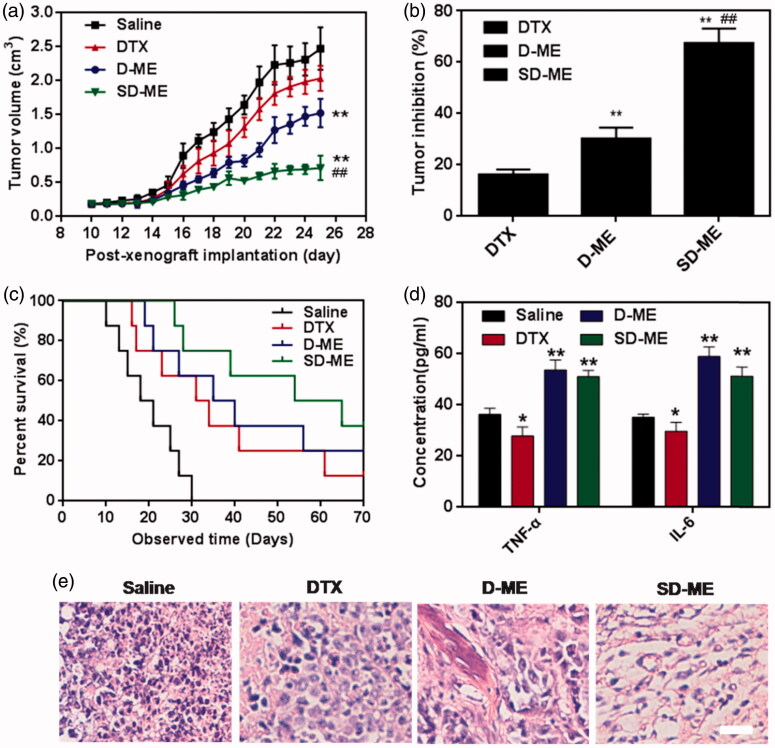
Evaluation on *in vivo* antitumor. (a) Changes in tumor volume of EC109/DDR tumor xenograft-bearing nude mice from day 10 to day 25. (*n* = 8, **p < 0.01 versus saline, ##*p *< 0.01 versus D-ME) (b) Tumor inhibition rate after antitumor treatment. (*n* = 8, **p < 0.01 versus DTX, ##*p *< 0.01 versus D-ME) (c) Survival curves within 70 days post-xenograft implantation. (d) TNF-α and IL-6 levels after intragastric administration of the different formulations for 14 days. (*n* = 4, *p < 0.05 and **p < 0.01 versus saline) (e) Histological section of tumor tissue collected at 72 h after the end of the treatment. The organs sections were stained with hematoxylin and eosin. Scale bar is 10 μm.

When the tumor of EC109/DDR xenograft-bearing nude mice grew to 80∼100 mm^3^, the *in vivo* antitumor efficacy was investigated through intragastric administration of DTX, D-ME, and SD-ME at a DTX concentration of 10 mg/kg once every day. As shown in Figure 5(a), the tumor size grew quickly during the period of treatment. At post-xenograft implantation day 25, the volume of tumors was nearly 23-fold larger than that at day 10. As a positive control, DTX did not effectively retard the growth of tumor size, with no significant difference as compared with the saline group. Several nanomedicines have been shown to overcome the insensitivity of tumor to chemotherapeutic agents through different mechanisms, including utilizing distinctive pathways for internalization and surfactant-mediated tight junction opening (Engle et al., 1995; Shubber et al., 2015). In a previous study, D-ME exhibited distinct advantages over DTX in the context of cytotoxicity, cellular uptake, and cell apoptosis. After therapy with D-ME for 14 days, the tumor size of nude mice decreased by 2-fold in comparison to saline group, and was significantly smaller than that in DTX group, indicative of the beneficial effects of microemulsion formulation. SD-ME had the most dominant and striking suppression of tumor growth among all the formulations. The size of tumor in SD-ME group was 5-fold and 4-fold smaller than saline and DTX group, respectively. Interestingly, the physical mixture of S + D was not capable of achieving such antitumor performance (data not shown). These findings further suggested that the rational drug combination and their tumor-oriented co-delivery were the two critical factors for the enhancement of *in vivo* antitumor efficacy observed for SD-ME. The tumor inhibition rate of SD-ME was 67.6 ± 5.6% (Figure 5b), even higher than some intravenously administered nanomedicines. Logically, the SD-ME-treated nude mice had the longest survival time in all treated groups. At the end of the observed time, the survival ratios of SD-ME and D-ME groups were 37.5% and 25%, respectively. Besides, the median survival times of DTX, D-ME, and SD-ME-treated mice were 31 days, 35 days, and 54 days, respectively (Figure 5c). It could be inferred that SD-ME can not only effectively suppress the tumor size, but also potentially prolong the life span of patients with drug-resistant esophageal carcinoma. Furthermore, we also observed the slightly elevated levels of TNF-α and IL-6 in plasma after treatment with D-ME and SD-ME (Figure 5d). It was probably connected with the enhanced immunomodulatory activities (Li et al., 2016). In order to investigate the morphological changes in the inner side of tumor, the tumor tissues were harvested, followed by cryostat section and staining with hematoxylin and eosin at 72 h after the ending of the treatment. As shown in Figure 5(e), cancer cells remission was obviously found in the tumor tissue after treatment with SD-ME, further proving the most prominent therapeutic activity *in vivo*.

### Safety evaluation *in vivo*

**Figure 6.**
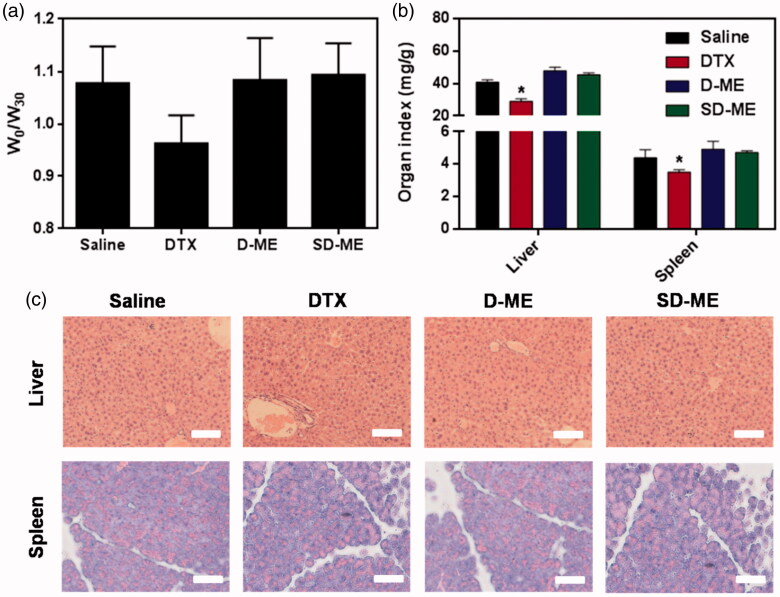
(a) The ratio of body weight between day 0 and day 30. (b) The weight ratio of liver/spleen to body at 72 h after the last administration. (*p < 0.05 versus saline, *n* = 4) (c) Histological section of liver and spleen collected at 72 h after the end of the treatment. The organs sections were stained with hematoxylin and eosin. Scale bar is 20 μm.

To assess the *in vivo* safety of treatment with various formulations, we monitored the body weight during the therapy and evaluated the liver and spleen (the most susceptible tissues) index at day 30. As shown in Figure 6(a), there was no significant decrease in body weight in D-ME and SD-ME treatment groups compared to that for the negative control group. However, the body weight in DTX treatment group at day 30 showed a mild reduction relative to that at day 0 although no statistical difference was found. The absence of changes in body weight indicated that SD-ME at this determined dose had no obvious systemic toxicity within the time period of the treatment. To assess the toxicity of DTX and the two types of microemulsion against susceptible tissues, we collected and weighed the liver and the spleen. As shown in Figure 6(b), the results demonstrated that DTX resulted in a significant decrease in the weight of the liver and the spleen compared with saline, suggesting a potential immunologic suppression. In contrast, the liver and spleen index in SD-ME and D-ME groups was similar to that in the saline group, indicating an apparent reduction of immunotoxicity. Besides, tissue histopathologic assessment was also performed to detect any potential lesion of the liver and the spleen. As shown in Figure 6(c), we did not observe any evident solid lesion or abnormality in the H&E-stained pathological sections, suggesting that SD-ME could be safe as an oral anticancer drug delivery system *in vivo*.

## Conclusion

In this study, we developed a microemulsion-based co-delivery system simultaneously carrying DTX and Schizandrin A, for the improvement of treatment of drug-resistant esophageal carcinoma. SD-ME had a small size and a mildly negative charge, as well as a high DEE and acceptable stability. SD-ME could promote the EC109/DDR cellular uptake and enhance the cytotoxicity in EC109/DDR cells. The mechanism of anti-drug resistance was elucidated as the inhibition of P-gp ATPase activity and the reduction of P-gp expression. Importantly, SD-ME exhibited a potent inhibition of tumor size and an overwhelming increase in the survival time among all the treatment groups, with no obvious toxicity against major normal tissues. Further evaluation of the mechanism of SD-ME’s antitumor efficacy *in vivo* is being undertaken by our group.
